# Association Between Combined Preoperative Alkaline Phosphatase-Lactate Dehydrogenase Status and Postoperative Outcomes After Pancreaticoduodenectomy for Periampullary Carcinoma

**DOI:** 10.7759/cureus.113383

**Published:** 2026-07-26

**Authors:** Md Abdulla Al Mansur, Mohammad Saief Uddin, Md Abdullah-Hel Azom, Emran Ali, Sabuj Kumar Patra, Kamrul A Hillol, Amit Chowdhury, Muhammad Salauddin, Shah Md Ezaz-ul Haque, Bidhan C Das

**Affiliations:** 1 Department of Hepatobiliary, Pancreatic and Liver Transplant Surgery, Bangladesh Medical University, Dhaka, BGD; 2 Department of Surgery, Directorate General of Health Services, Dhaka, BGD; 3 Department of Hepatobiliary Surgery, United Medical College Hospital, Dhaka, BGD; 4 Department of Public Health, Dhaka Medical College, Dhaka, BGD

**Keywords:** alkaline phosphatase, lactate dehydrogenase, pancreaticoduodenectomy, periampullary carcinoma, postoperative complications

## Abstract

Background: Periampullary carcinoma is a heterogeneous group of tumours arising within 2 cm of the papilla of Vater, including pancreatic, ampullary and biliary cancers, and is most commonly treated with pancreaticoduodenectomy (PD), a complex procedure associated with substantial postoperative morbidity. The combination of alkaline phosphatase (ALP) and lactate dehydrogenase (LDH) provides more information than either marker individually regarding hepatobiliary tumour burden and tumour metabolic activity, respectively. This study aimed to examine the association between combined ALP-LDH status and the postoperative outcome of patients with periampullary carcinoma after PD.

Methods: This was a prospective, observational study in the Department of Hepatobiliary, Pancreatic and Liver Transplantation Surgery, Bangladesh Medical University, Dhaka, from December 2024 till November 2025. Thirty periampullary carcinoma patients admitted for PD were enrolled consecutively. Patients were divided into three groups: Group 1 (ALP and LDH below their respective cut-off values, n=2); Group 2 (either ALP or LDH was elevated, n=10); and Group 3 (ALP and LDH both elevated, n=18). One-way ANOVA/Kruskal-Wallis and chi-square/Fisher-Freeman-Halton exact tests were used with effect sizes to compare groups with respect to the following variables: demographic, clinical, laboratory, tumour-related, operative, and postoperative variables. Complications after surgery were classified according to the Clavien-Dindo system.

Results: The mean age was 51.70 ± 11.03 years, and baseline demographic and clinical features were similar for the three groups. Of all the pre-operative laboratory parameters, only carbohydrate antigen 19-9 (CA 19-9) was significantly different: median 26.48 U/mL in Group 1, 212.40 U/mL in Group 2 and 569.40 U/mL in Group 3 (epsilon squared = 0.208, P = 0.022); the other laboratory parameters did not show a significant difference. The sizes of the tumour, nodal status, operative time, blood loss and pancreatic fistulas were not significantly correlated with ALP-LDH status. The major complications (Clavien-Dindo grade ≥III) were increasing, but not statistically significantly, from 0% to 30.0% and 66.7% in Group 2 and Group 3, respectively. With regard to the postoperative outcomes, wound infection was the only one that was statistically significantly associated with combined ALP-LDH status (Cramer's V=0.601, P=0.004), with a rate of 0% in Group 1, 30.0% in Group 2 and 83.3% in Group 3.

Conclusion: In this small, exploratory cohort, preoperative ALP and LDH status were associated with the preoperative CA 19-9 level and postoperative wound infection, with a non-significant trend toward increased major complications; there was no significant association with most other postoperative outcomes such as pancreatic fistula, delayed gastric emptying, and 30-day mortality. Given the limited and unevenly distributed sample, these findings should be regarded as hypothesis-generating and require validation in larger, multicentre cohorts before any clinical application.

## Introduction

Periampullary carcinoma is a complex disease of heterogeneous origin that arises within 2 cm of the papilla of Vater and includes pancreatic, ampullary, biliary and duodenal cancers [1]. Although these tumours are located in a close anatomical area, they have different biology, surgical risks and postoperative outcomes [2]. The Whipple procedure, also known as pancreaticoduodenectomy (PD), is a mainstay of treatment for resectable periampullary carcinoma, which is still a very complex procedure with early mortality of less than 5% and postoperative morbidity of 40%-50%. But there have been some excellent advances in surgical techniques and specialised peri-operative care [3]. Despite advancements in anaesthetic technique, operative management and peri-operative care, there is significant morbidity following PD, which can include postoperative pancreatic fistula (POPF), delayed gastric emptying (DGE), post-pancreatectomy haemorrhage (PPH), bile leak, wound infection and longer hospital stay [4, 5]. Therefore, the clinical significance of identifying patients at higher risk of developing adverse postoperative outcomes is evident in surgical planning, patient counselling, perioperative management and allocation of postoperative monitoring resources. There are several scoring systems developed to predict the major complications after PD, but due to various reasons, the use of these scores for prediction in practice is limited [6].

A simple, low-cost and readily available biomarker preoperatively could be especially useful in resource-limited settings. Biochemical parameters are emerging as prognostic markers of cancer due to the fact that these can be easily measured and can reflect tumour progression, biliary obstruction, systemic inflammation, cellular proliferation and altered tumour metabolism, which may happen preoperatively or peri-operatively [7, 8]. Alkaline phosphatase (ALP) is a hydrolase enzyme, which is found in hepatobiliary tissue and is elevated in biliary obstruction and liver involvement with the biological activity of a tumour. There have been reports of pancreatic carcinoma-associated ALP expression, and high ALP levels have been associated with poor prognosis in various malignancies such as hepatocellular, colorectal, nasopharyngeal, prostate and oesophageal cancers [9-16]. Lactate dehydrogenase (LDH) is a key enzyme in anaerobic glycolysis and reflects the metabolic reprogramming that supports tumour growth, tissue hypoxia and aggressive cancer biology. Raised LDH has been associated with tumour burden and poorer prognosis in different cancers, and experimental and clinical studies support its relevance to malignant progression [17-19]. Importantly, the combined assessment of ALP and LDH might prove to provide more comprehensive information than either signal alone, since these parameters might also provide insight into hepatobiliary tumour burden and hepatobiliary metabolic activity. In pancreatic ductal adenocarcinoma, combined preoperative ALP and LDH levels have been associated with disease-free and overall survival, while a large periampullary carcinoma cohort found that elevated ALP and LDH were independent prognostic factors after PD [7, 20].

Though evidence is yet limited in Bangladesh, the clinical burden of periampullary and biliary tract malignancies with their aggressive nature is emerging from the available local data [21]. Accordingly, evaluating combined preoperative ALP and LDH status may help identify high-risk patients before PD and support individualised perioperative decision-making. This study aimed to determine the association between combined preoperative ALP-LDH status and short-term postoperative morbidity and mortality occurring within 30 days after pancreaticoduodenectomy in patients with periampullary carcinoma.

## Materials and methods

Study design and setting

This was a prospective observational study conducted in the Department of Hepatobiliary, Pancreatic and Liver Transplantation Surgery, Bangladesh Medical University (BMU), Shahbag, Dhaka, Bangladesh, from December 2024 to November 2025, following approval of the study protocol by the Institutional Review Board (IRB) of BMU.

Study participants

All patients who underwent PD during the study period in the Department of Hepatobiliary, Pancreatic and Liver Transplantation Surgery, BMU, Dhaka, after fulfilling inclusion and exclusion criteria. In the American Society of Anaesthesiologists (ASA) physical status classification, ASA I refers to a completely healthy and fit patient with no systemic disease, and ASA II refers to a patient with mild systemic disease [22]. Enrolment criteria are described in Table 1.

**Table 1:** Enrolment criteria for study participants

Inclusion criteria	Exclusion criteria
Adult patients aged 18-75 years undergoing pancreaticoduodenectomy for periampullary carcinoma	Evidence of distant metastasis
American Society of Anesthesiologists (ASA) physical status I-II	Severe hepatic dysfunction unrelated to biliary obstruction
Available preoperative alkaline phosphatase (ALP) and lactate dehydrogenase (LDH) values	Incomplete core data or refusal of consent
Patient with or without endoscopic retrograde cholangiopancreatography (ERCP) stenting	Unresectable disease or palliative bypass only

Sample size

For the sample size, the Krejcie and Morgan finite population correction formula was used [23]. The formula is:

$n = \frac{\chi^2 N P (1 - P)}{e^2 (N - 1) + \chi^2 P (1 - P)}$

Here, considering population size (N = 32) based on the number of PD procedures performed in the department during the last 12 months at BMU, a 5% acceptable sampling error (e), a chi-square value (χ²) of 3.841 for one degree of freedom at the 95% confidence level and an assumed population proportion (P) of 0.5 (the most conservative assumption, maximising the estimated variance in the absence of prior local prevalence data), the calculated minimum sample size (n) was approximately 29.6, rounded up to 30 patients. This calculation was used only to confirm the feasibility of enrolling an adequate number of patients from the available annual departmental caseload (N=32) and did not constitute a formal a priori power calculation for the three-group ALP-LDH comparison; no such power calculation was performed, given the exploratory, hypothesis-generating design of the study and the anticipated imbalance in group sizes based on the expected distribution of ALP-LDH status in this population. All eligible patients presenting during the study period were enrolled by a consecutive sampling method until the target minimum sample size was reached.

Study variables

Independent variables included demographic data (age and sex), clinical variables (body mass index, preoperative weight loss, comorbidities, ASA score, and preoperative diagnosis), and laboratory variables (haemoglobin, total leukocyte count, platelet count, total bilirubin, serum glutamate pyruvate transaminase (SGPT), serum glutamic-oxaloacetic transaminase (SGOT), serum albumin, and carbohydrate antigen 19-9 (CA 19-9)).

Cutoff determination of LDH/ALP and grouping

Preoperative serum ALP and lactate LDH were measured using a Cobas C311 (Roche, Germany) automated biochemistry analyser from venous blood samples collected and processed under standardised laboratory conditions in the Department of Laboratory Medicine, BMU. At a cutoff value of 263 U/L, preoperative ALP demonstrated sensitivity of 84.2% and specificity of 27.3% for predicting postoperative outcome following pancreaticoduodenectomy, with an AUC of 0.770 (95% CI: 0.601-0.939), showing statistical significance (P=0.015). At a cutoff value of 175.5 U/L, preoperative LDH showed sensitivity of 89.5% and specificity of 45.5% in predicting postoperative outcome after pancreaticoduodenectomy, with an AUC of 0.756 (95% CI: 0.566-0.946), reaching statistical significance (P=0.021). So, the cutoff values of ALP and LDH were 263 and 175.5 U/L, respectively. An increase in serum enzyme level was considered to be having a serum enzyme level above the cutoff. Like other studies, patients were stratified into three groups: Group 1 (both ALP and LDH low), Group 2 (either ALP or LDH elevated), and Group 3 (both ALP and LDH elevated) [7, 20].

Outcome variables

Outcome variables comprised preoperative parameters (duration of surgery, intraoperative blood loss, and blood transfusion requirement) and postoperative parameters, including histopathological findings (tumour size, histologic grade, resection margin status, perineural and lymphovascular invasion), POPF, DGE, bile leakage, wound infection, postoperative hospital stay, and 30-day mortality. Postoperative complications were graded using the Clavien-Dindo classification system, where Grade I was a minor deviation from normal postoperative recovery which did not require significant medical/surgical/endoscopic/imaging treatment. In this grade, there were simple treatments like painkillers, fever treatments, anti-sickness drugs, electrolytes, diuretics and physiotherapy. Grade II complications were more serious and involved the use of drugs other than the basic drugs permitted in grade II. This category also covered those cases that necessitated blood transfusion and total parenteral nutrition. Grade III complications needed an ongoing intervention, such as a surgical, endoscopic or radiological procedure. There were two subgroups to this grade condition: Grade IIIa, which was an intervention performed without general anaesthesia, and Grade IIIb, which was an intervention performed with general anaesthesia. Grade IV - life-threatening complications, which needed to be managed in an intermediate care or intensive care unit. It was also subdivided into two subgroups: Grade IVa, single-organ dysfunction (including those needing dialysis); and Grade IVb, multiorgan dysfunction. Grade V was the most severe, which indicates the death of the patient [24].

POPF was defined as a complication of pancreatic surgery that resulted in a leakage of pancreatic fluid where there was an abnormal communication between the pancreatic ductal epithelium and another epithelial surface, and the pancreatic leak should have also resulted in a clinically significant issue or a change in patient management. It was graded as an asymptomatic pancreatic leak without clinical impact, and clinically relevant grades were Grade B (when the pancreatic leak necessitated postoperative changes in treatment, including the use of antibiotics, nutritional support, percutaneous drainage, endoscopic drainage or angiographic treatment) and Grade C (which occurred when the fistula caused organ failure, reoperation or death) [25]. DGE was defined as a postoperative complication characterised by the stomach not emptying normally after surgery. DGE is categorised into three grades depending on the length of time the nasogastric tube was required and the time before they were able to tolerate solid food. DGE (grade A) required a nasogastric tube for 4-7 days or reinsertion after postoperative day 3, and no solid food was tolerated by postoperative day 7. Grade B was moderate DGE, requiring the use of a tube for 8-14 days or reinsertion after postoperative day 7 and not tolerating solids by postoperative day 14. Severe DGE (Grade C) was defined as being in place for over 14 days or reinserted after day 14 of surgery and the patient still not eating solid food by day 21 [26].

Data collection procedure

All biochemical investigations were performed in the Department of Laboratory Medicine, BMU, using a Cobas C311 (Roche, Germany) automated biochemistry analyser. Preoperative biochemical investigations were performed within 72 hours before pancreaticoduodenectomy. A sample of 2-3 ml of blood collected in a red tube was sent to the Department of Laboratory Medicine within 15 minutes of collection. After receiving the tube, it was labelled with a barcode for patient identification. Blood was allowed to clot for 15 to 20 minutes at room temperature. It was then centrifuged at a rate of 3000 rpm for 10 minutes, following which serum was separated carefully into a clean tube. After separation of serum, the sample was placed in an automated spectrophotometric machine (Cobas C311). The machine aspirated and sampled automatically. The analyser calculated LDH and ALP activity in U/L. Before taking informed written consent, all the procedures and risks and benefits of the study were explained to the patients. Conventional PD was done. Confidentiality of the patients was maintained. Data were collected using a pre-structured checklist covering patient records, clinical history, physical examination, preoperative laboratory and imaging findings, intraoperative observations, and postoperative outcomes up to 30 days after surgery, including outpatient follow-up between postoperative days 14 and 30. All data were collected by the investigator following written informed consent obtained in the local language after explaining the study procedure, risks, and benefits to participants.

Ethical considerations

Ethical approval was obtained from the Institutional Review Board of Bangladesh Medical University (BMU) (formerly Bangabandhu Sheikh Mujib Medical University), Dhaka, Bangladesh (Approval No. BSMMU/2024/10711(47), dated November 26, 2024) prior to the commencement of the study. Confidentiality and anonymity of all participants were maintained throughout data collection, storage, and analysis, and non-participation did not affect the quality of clinical care provided.

Statistical analysis

Data were processed and analysed using IBM SPSS Statistics for Windows, Version 27.0 (IBM Corp., Armonk, NY). Continuous variables were expressed as mean ± standard deviation or median with interquartile range (IQR), as appropriate, and compared among the three combined ALP-LDH groups using one-way ANOVA or the Kruskal-Wallis test, with eta-squared or epsilon-squared reported as effect size measures. Categorical variables were expressed as frequencies and percentages and compared using Pearson's chi-square test; the Fisher-Freeman-Halton (FFH) exact test was applied for sparse contingency tables, with Cramer's V reported as the effect size. ALP and LDH values were not statistically tested as outcome variables since they were used to define the exposure groups. A p-value of less than 0.05 was considered statistically significant, with the confidence interval set at 95%. Multivariable regression modelling was not performed, as the small overall sample size (N=30) and the pronounced imbalance across ALP-LDH strata (n=2, 10, and 18) would have resulted in an inadequate number of events per variable and unstable effect estimates; this is acknowledged as a limitation, and findings from univariable comparisons should accordingly be interpreted as hypothesis-generating rather than confirmatory.

## Results

A total of 30 patients with periampullary carcinoma undergoing pancreaticoduodenectomy were included. Using ROC-derived cutoff values of 263 U/L for ALP and 175.5 U/L for LDH, patients were categorised as Group 1, with both ALP and LDH low (n=2); Group 2, with either ALP or LDH elevated (n=10); and Group 3, with both ALP and LDH elevated (n=18). The overall mean +/- SD age was 51.70 +/- 11.03 years, with no significant difference across groups (F(2, 27) = 0.001, eta squared = 0.000, P = 0.999). Age groups, sex, body mass index (BMI), preoperative weight loss, comorbidities, ASA score, and preoperative diagnosis were also comparable among the groups. The most common preoperative diagnosis was ampullary carcinoma (36.7%), followed by carcinoma of the head of the pancreas and distal bile duct carcinoma (26.7% each) (Table 2).

**Table 2:** Baseline demographic and clinical characteristics by combined ALP-LDH status (N=30) ALP: alkaline phosphatase; LDH: lactate dehydrogenase; BMI: body mass index; ASA: American Society of Anesthesiologists; SD: standard deviation; ANOVA: analysis of variance; FFH: Fisher-Freeman-Halton exact test

Characteristic	Total (N=30)	Group 1 (n=2)	Group 2 (n=10)	Group 3 (n=18)	Test used / statistic	Effect size	P-value
Age, years, mean ±SD	51.70 ± 11.03	52.00 ± 2.83	51.60 ± 9.56	51.72 ± 12.60	One-way ANOVA F(2,27)=0.001	eta squared=0.000	0.999
Age group, n (%)							
≤30	2 (6.7)	0 (0.0)	0 (0.0)	2 (11.1)	FFH exact; Pearson chi-square(8)=3.859	Cramer’s V=0.254	0.930
31-40	3 (10.0)	0 (0.0)	1 (10.0)	2 (11.1)			
41-50	11 (36.7)	1 (50.0)	5 (50.0)	5 (27.8)			
51-60	7 (23.3)	1 (50.0)	2 (20.0)	4 (22.2)			
61-70	7 (23.3)	0 (0.0)	2 (20.0)	5 (27.8)			
Sex, n (%)							
Male	17 (56.7)	1 (50.0)	7 (70.0)	9 (50.0)	FFH exact; Pearson chi-square(2)=1.086	Cramer’s V=0.190	0.713
Female	13 (43.3)	1 (50.0)	3 (30.0)	9 (50.0)			
BMI, kg/m^2^, mean ±SD	20.93 ± 4.15	22.70 ± 2.69	21.99 ± 5.52	20.14 ± 3.33	One-way ANOVA F(2,27)=0.826	eta squared=0.058	0.448
Preoperative weight loss, kg, mean ±SD	4.20 ± 2.81	1.00 ± 1.41	3.70 ± 2.50	4.83 ± 2.87	One-way ANOVA F(2,27)=2.053	eta squared=0.132	0.148
Diabetes mellitus, present, n (%)	13 (43.3)	0 (0.0)	6 (60.0)	7 (38.9)	FFH exact; Pearson chi-square(2)=2.805	Cramer’s V=0.306	0.373
Hypertension, present, n (%)	7 (23.3)	0 (0.0)	1 (10.0)	6 (33.3)	FFH exact; Pearson chi-square(2)=2.609	Cramer’s V=0.295	0.399
Ischemic heart disease, present, n (%)	1 (3.3)	0 (0.0)	1 (10.0)	0 (0.0)	FFH exact; Pearson chi-square(2)=2.069	Cramer’s V=0.263	0.400
Chronic obstructive pulmonary disease, present, n (%)	2 (6.7)	0 (0.0)	1 (10.0)	1 (5.6)	FFH exact; Pearson chi-square(2)=0.357	Cramer’s V=0.109	1.000
Asthma, present, n (%)	1 (3.3)	0 (0.0)	1 (10.0)	0 (0.0)	FFH exact; Pearson chi-square(2)=2.069	Cramer’s V=0.263	0.400
Chronic kidney disease, present, n (%)	1 (3.3)	0 (0.0)	1 (10.0)	0 (0.0)	FFH exact; Pearson chi-square(2)=2.069	Cramer’s V=0.263	0.400
ASA score, n (%)							
ASA I	16 (53.3)	1 (50.0)	6 (60.0)	9 (50.0)	FFH exact; Pearson chi-square(2)=0.268	Cramer’s V=0.094	0.848
ASA II	14 (46.7)	1 (50.0)	4 (40.0)	9 (50.0)			
Preoperative diagnosis, n (%)							
Carcinoma head of pancreas	8 (26.7)	0 (0.0)	3 (30.0)	5 (27.8)	FFH exact; Pearson chi-square(6)=5.571	Cramer’s V=0.305	0.670
Distal bile duct carcinoma	8 (26.7)	0 (0.0)	3 (30.0)	5 (27.8)			
Duodenal carcinoma	3 (10.0)	1 (50.0)	0 (0.0)	2 (11.1)			
Ampullary carcinoma	11 (36.7)	1 (50.0)	4 (40.0)	6 (33.3)			

Preoperative laboratory and tumour-marker profile

Preoperative laboratory findings are shown in Table 3. Haemoglobin, total leukocyte count, platelet count, serum albumin, and carcinoembryonic antigen (CEA) did not differ significantly across the three ALP-LDH groups. Median total bilirubin increased from 0.48 mg/dL in Group 1 to 8.64 mg/dL in Group 2 and 10.00 mg/dL in Group 3, but this difference was not statistically significant (P=0.059). SGPT and SGOT were higher in the ALP-LDH-elevated group but remained non-significant (P=0.089 and P=0.104, respectively). CA 19-9 differed significantly across groups, with median values of 26.48, 212.40, and 569.40 U/mL in Groups 1, 2, and 3, respectively (H(2)=7.617, epsilon squared=0.208, P=0.022) (Table 3).

**Table 3:** Preoperative laboratory and tumour-marker profile by combined ALP-LDH status (N=30) ALP: alkaline phosphatase; LDH: lactate dehydrogenase; SD: standard deviation; IQR: interquartile range; SGPT: serum glutamic pyruvate transaminase; SGOT: serum glutamic-oxaloacetic transaminase; CA 19-9: carbohydrate antigen 19-9; CEA: carcinoembryonic antigen; ANOVA: analysis of variance

Characteristic	Total (N=30)	Group 1 (n=2)	Group 2 (n=10)	Group 3 (n=18)	Test used/statistic	Effect size	P-value
Haemoglobin, g/dL, mean ± SD	10.48 ± 2.33	11.60 ± 0.71	10.70 ± 2.45	10.23 ± 2.42	One-way ANOVA F(2,27)=0.3577	eta squared=0.0258	0.703
Total leukocyte count, cells/mm^3^, mean ± SD	10167.47 ± 3063.89	9000.00 ± 707.11	9736.20 ± 3622.96	10536.78 ± 2936.20	One-way ANOVA F(2,27)=0.3584	eta squared=0.0259	0.702
Platelet count, cells/uL, median (IQR)	357858.00 (274806.75-510182.75)	321500.00 (315750.00-327250.00)	357858.00 (277750.00-509750.00)	451556.00 (259806.75-510182.75)	Kruskal-Wallis H(2)=0.276	epsilon squared=0.000	0.871
Total bilirubin, mg/dL, median (IQR)	8.97 (3.36-13.17)	0.48 (0.34-0.63)	8.64 (1.12-12.35)	10.00 (7.11-13.47)	Kruskal-Wallis H(2)=5.672	epsilon squared=0.136	0.059
SGPT, U/L, median (IQR)	74.55 (45.83-122.90)	14.20 (13.85-14.55)	67.50 (28.23-137.00)	94.00 (54.98-115.45)	Kruskal-Wallis H(2)=4.847	epsilon squared=0.105	0.089
SGOT, U/L, median (IQR)	45.95 (24.18-65.58)	16.95 (15.97-17.92)	45.95 (22.00-53.83)	51.55 (33.27-82.00)	Kruskal-Wallis H(2)=4.521	epsilon squared=0.093	0.104
Serum albumin, g/dL, mean ± SD	3.69 ± 1.15	3.43 ± 0.87	3.87 ± 0.90	3.62 ± 1.33	One-way ANOVA F(2,27)=0.194	eta squared=0.014	0.825
CA 19-9, U/mL, median (IQR)	382.50 (126.75-882.00)	26.48 (20.09-32.86)	212.40 (81.00-365.25)	569.40 (215.00-1000.00)	Kruskal-Wallis H(2)=7.617	epsilon squared=0.208	0.022
CEA, ng/mL, median (IQR)	3.98 (2.10-76.21)	2.14 (2.10-2.17)	4.35 (3.13-85.42)	4.68 (1.73-76.21)	Kruskal-Wallis H(2)=1.669	epsilon squared=0.000	0.434

Tumour-related and operative variables

Tumour-related and operative variables are summarised in Table 4. Tumour size was evenly distributed, with 15 patients (50.0%) having tumours <3 cm and 15 patients (50.0%) having tumours ≥3 cm (P=0.842). Although T3 disease was more frequent in Group 3 (50.0%) and all Group 1 patients had T1 disease, depth of tumour was not significantly associated with ALP-LDH status (P = 0.090). Lymph node metastasis was present in 15 patients (50.0%) and was most frequent in Group 3 (61.1%), but the difference was not significant (P=0.233). All patients had M0 disease and underwent classic pancreaticoduodenectomy. The main pancreatic duct (MPD) diameter, common bile duct (CBD) diameter, operative duration, blood loss, and transfusion requirement were similar across groups. Pancreatic texture showed a non-significant trend (P = 0.077) (Table 4).

**Table 4:** Tumour-related, operative, and postoperative variables by combined ALP-LDH status (N=30) ALP: alkaline phosphatase; LDH: lactate dehydrogenase; PD: pancreaticoduodenectomy; MPD: main pancreatic duct; CBD: common bile duct; POPF: postoperative pancreatic fistula; DGE: delayed gastric emptying; SD: standard deviation; IQR: interquartile range; ANOVA: analysis of variance; FFH: Fisher-Freeman-Halton exact test

Characteristic	Total (N=30)	Group 1 (n=2)	Group 2 (n=10)	Group 3 (n=18)	Test used/statistic	Effect size	P-value
Tumour size, n (%)							
<3 cm	15 (50.0)	1 (50.0)	6 (60.0)	8 (44.4)	FFH exact; Pearson chi-square(2)=0.622	Cramer’s V=0.144	0.842
≥3 cm	15 (50.0)	1 (50.0)	4 (40.0)	10 (55.6)			
Depth of tumour, n (%)							
T1	7 (23.3)	2 (100.0)	2 (20.0)	3 (16.7)	FFH exact; Pearson chi-square(4)=9.792	Cramer’s V=0.404	0.090
T2	12 (40.0)	0 (0.0)	6 (60.0)	6 (33.3)			
T3	11 (36.7)	0 (0.0)	2 (20.0)	9 (50.0)			
T4	0 (0.0)	0 (0.0)	0 (0.0)	0 (0.0)			
Lymph node metastasis, n (%)							
N0	15 (50.0)	2 (100.0)	6 (60.0)	7 (38.9)	FFH exact; Pearson chi-square(2)=3.289	Cramer’s V=0.331	0.233
N1	15 (50.0)	0 (0.0)	4 (40.0)	11 (61.1)			
N2	0 (0.0)	0 (0.0)	0 (0.0)	0 (0.0)			
Distant metastasis, n (%)							
M0	30 (100.0)	2 (100.0)	10 (100.0)	18 (100.0)	-	-	-
MPD diameter, mm, median (IQR)	5.00 (3.25-6.00)	5.00 (4.00-6.00)	4.00 (3.25-5.00)	5.00 (4.00-6.00)	Kruskal-Wallis H(2)=1.171	epsilon squared=0.000	0.557
CBD diameter, mm, median (IQR)	15.50 (12.25-18.93)	16.00 (14.00-18.00)	14.00 (9.25-15.75)	17.50 (13.25-19.75)	Kruskal-Wallis H(2)=3.211	epsilon squared=0.045	0.201
Duration of surgery, min, mean ± SD	376.60 ± 55.40	385.00 ± 49.50	375.80 ± 58.49	376.11 ± 57.19	One-way ANOVA F(2,27)=0.023	eta squared=0.002	0.977
Blood loss, mL, mean ± SD	257.63 ± 140.09	350.00 ± 282.84	228.80 ± 117.14	263.39 ± 141.35	One-way ANOVA F(2,27)=0.646	eta squared=0.046	0.532
Blood transfusion, units, mean ± SD	2.13 ± 1.04	2.50 ± 2.12	2.00 ± 1.05	2.17 ± 0.99	One-way ANOVA F(2,27)=0.203	eta squared=0.015	0.817
Operation method, n (%)							
Classic PD	30 (100.0)	2 (100.0)	10 (100.0)	18 (100.0)	-	-	-
Pancreas texture, n (%)							
Soft	12 (40.0)	0 (0.0)	2 (20.0)	10 (55.6)	FFH exact; Pearson chi-square(4)=9.006	Cramer’s V=0.387	0.077
Moderate	10 (33.3)	2 (100.0)	3 (30.0)	5 (27.8)			
Hard	8 (26.7)	0 (0.0)	5 (50.0)	3 (16.7)			
Postoperative hospital stay, days, mean ± SD	19.60 ± 6.00	18.00 ± 1.41	19.20 ± 5.73	20.00 ± 6.58	One-way ANOVA F(2,27)=0.125	eta squared=0.009	0.883
Any complication, present, n (%)	21 (70.0)	0 (0.0)	6 (60.0)	15 (83.3)	FFH exact; Pearson chi-square(2)=6.667	Cramer’s V=0.471	0.045
Clavien-Dindo classification, n (%)							
No complication	9 (30.0)	2 (100.0)	4 (40.0)	3 (16.7)	FFH exact; Pearson chi-square(14)=13.444	Cramer’s V=0.473	0.404
Grade I	2 (6.7)	0 (0.0)	1 (10.0)	1 (5.6)			
Grade II	4 (13.3)	0 (0.0)	2 (20.0)	2 (11.1)			
Grade IIIa	6 (20.0)	0 (0.0)	0 (0.0)	6 (33.3)			
Grade IIIb	3 (10.0)	0 (0.0)	1 (10.0)	2 (11.1)			
Grade IVa	2 (6.7)	0 (0.0)	0 (0.0)	2 (11.1)			
Grade IVb	1 (3.3)	0 (0.0)	1 (10.0)	0 (0.0)			
Grade V	3 (10.0)	0 (0.0)	1 (10.0)	2 (11.1)			
Major complication, Clavien-Dindo ≥III	15 (50.0)	0 (0.0)	3 (30.0)	12 (66.7)	FFH exact; Pearson chi-square(2)=5.600	Cramer’s V=0.432	0.079
POPF, n (%)							
No fistula	12 (40.0)	1 (50.0)	6 (60.0)	5 (27.8)	FFH exact; Pearson chi-square(4)=5.220	Cramer’s V=0.295	0.222
Biochemical leak	5 (16.7)	1 (50.0)	1 (10.0)	3 (16.7)			
Grade B	13 (43.3)	0 (0.0)	3 (30.0)	10 (55.6)			
Grade C	0 (0.0)	0 (0.0)	0 (0.0)	0 (0.0)			
DGE, n (%)							
Grade A	10 (33.3)	2 (100.0)	4 (40.0)	4 (22.2)	Fisher-Freeman-Halton exact; Pearson chi-square(4)=5.285	Cramer's V=0.297	0.396
Grade B	11 (36.7)	0 (0.0)	3 (30.0)	8 (44.4)			
No DGE	9 (30.0)	0 (0.0)	3 (30.0)	6 (33.3)			
Bile leakage, present, n (%)	6 (20.0)	0 (0.0)	3 (30.0)	3 (16.7)	FFH exact; Pearson chi-square(2)=1.250	Cramer’s V=0.204	0.768
Wound infection, present, n (%)	18 (60.0)	0 (0.0)	3 (30.0)	15 (83.3)	FFH exact; Pearson chi-square(2)=10.833	Cramer’s V=0.601	0.004
Other complication, n (%)							
Renal failure	3 (10.0)	0 (0.0)	1 (10.0)	2 (11.1)	FFH exact; Pearson chi-square(8)=3.322	Cramer’s V=0.235	0.952
Pulmonary complications	4 (13.3)	0 (0.0)	2 (20.0)	2 (11.1)			
Hepatic failure	1 (3.3)	0 (0.0)	0 (0.0)	1 (5.6)			
Cardiac complications	2 (6.7)	0 (0.0)	0 (0.0)	2 (11.1)			
No/other	20 (66.7)	2 (100.0)	7 (70.0)	11 (61.1)			
30-day mortality, n (%)	3 (10.0)	0 (0.0)	1 (10.0)	2 (11.1)	FFH exact; Pearson chi-square(2)=0.247	Cramer’s V=0.091	1.000

Postoperative outcomes

Postoperative outcomes are presented in Table 4. The mean postoperative hospital stay was 19.60 ± 6.00 days and was not significantly different across groups (P = 0.883). Any postoperative complication occurred in 21 patients (70.0%), including 0.0% of Group 1, 60.0% of Group 2, and 83.3% of Group 3 (P = 0.045). The distribution of Clavien-Dindo grades was not significantly different (P=0.404). Major complications, defined as Clavien-Dindo grade III or higher, increased from 0.0% in Group 1 to 30.0% in Group 2 and 66.7% in Group 3, but this did not reach statistical significance (P=0.079). POPF was not associated with ALP-LDH status (P=0.222). DGE distribution was also similar across groups: Grade A occurred in 10 patients (33.3%), Grade B in 11 patients (36.7%), and no DGE in 9 patients (30.0%) (Fisher-Freeman-Halton exact test; Pearson chi-square (4)=5.285, Cramer's V=0.297, P=0.396). Bile leakage, other complications, and 30-day mortality were not significantly different. Wound infection was the only postoperative outcome significantly associated with combined ALP-LDH status, increasing from 0.0% in Group 1 to 30.0% in Group 2 and 83.3% in Group 3 (Pearson chi-square (2)=10.833, Cramer's V=0.601, P=0.004).

## Discussion

In the present study, the association between the combination of pre-operative alkaline ALP and LDH status and outcomes after PD for periampullary carcinoma was examined. There were several clinically important findings were observed. The overall age, sex, body mass index, comorbidities, ASA status, and preoperative diagnosis of the combined ALP-LDH groups were comparable and not significantly different. The level of CA 19-9 increased across the groups (P = 0.022) and was the only laboratory marker to be statistically significant. The levels of total bilirubin, SGPT and SGOT were also raised but not significantly. Of the postoperative variables, wound infection was the most obvious association with combined ALP-LDH status (P=0.004) and was the most common in the combined elevated ALP-LDH group. There was also a trend towards major complications that increased from Group 1 to Group 3, but this did not differ significantly (P=0.079).

These findings partly support another study where the authors analysed 185 cases of pancreatic ductal adenocarcinoma (PDAC) that were resectable; these authors found that high levels of LDH and ALP correlated with the markers CA 19-9, presence of lymph nodes, tumour size, TNM stage, recurrence and distant metastasis [20]. They found that LDH and ALP were independent predictors of disease-free and overall survival (DFS and OS) and that the model using both LDH and ALP had increasingly poor outcomes from low- to high-risk groups [20]. The periampullary cohort in the current study had a significant gradient of CA 19-9, and Group 3 had a higher periampullary wound infection burden, which are directionally similar to the adverse-risk pattern described by Ji et al. [20]. Given the number of variables compared across the three groups without adjustment for multiple testing, the statistically significant findings (CA 19-9, wound infection) should be interpreted with caution and considered hypothesis-generating. Unlike those found in the PDAC cohort, however, the present study did not reveal significant associations with the tumour size, nodal status, pancreatic fistula, delayed gastric emptying or 30-day mortality.

This difference is theoretically and technically possible. The biological explanation for the observed association between combined ALP-LDH elevation and postoperative wound infection remains hypothetical. Elevated ALP may reflect biliary obstruction, cholestasis, or hepatobiliary tumour burden, whereas elevated LDH may reflect systemic inflammation, tissue hypoxia, increased tumour metabolic activity, or cellular injury. Severe biliary obstruction and cholangitis may contribute to malnutrition, impaired immune function, bacterial colonisation of bile, and delayed wound healing, thereby increasing susceptibility to surgical-site infection. However, these mechanisms were not directly evaluated in the present study. The association may therefore be attributable partly or entirely to residual confounding by jaundice, cholangitis, biliary drainage, nutritional status, tumour subtype, or other perioperative factors rather than to an independent effect of ALP or LDH. In patients with hepatocellular carcinoma (HCC), a retrospective study showed that ALP, gamma-glutamyl transpeptidase (GGT) and LDH before liver resection were indicators of prognosis, while Li et al. reported that pretreatment serum LDH and ALP were poor prognosis factors in nasopharyngeal carcinoma (NPC) [13, 16]. Another study also demonstrated that an ALP-based ratio could predict the recurrence and survival following hepatectomy [27]. These studies have supported enzyme-based markers, but most of the studies examined long-term survival and not early postoperative morbidity.

The present study is also distinct from the periampullary survival from the study conducted by Maharjan et al., where pancreatic head carcinoma showed the worst survival, while lymphovascular invasion (LVI), perineural invasion (PNI), lymph node ratio (LNR), and nodal involvement were associated with poor median survival, and PNI was independently associated with poor median survival [3]. Tumour depth and nodal metastasis were both more common but not statistically significantly different between the groups in this analysis. This could be due to the small number of patients, unequal distribution of the groups, intra-cavity tumour heterogeneity, and the fact that only short-term postoperative outcomes were studied rather than survival. Additional evidence from other malignancies further strengthens the scientific rationale for including serum enzyme markers in perioperative risk interpretation. A meta-analysis of 10 osteosarcoma studies involving 943 patients reported that high serum LDH was associated with worse overall survival, with a pooled hazard ratio of 1.92 (95% CI, 1.53-2.40), supporting LDH as a reproducible marker of adverse tumour biology across cancer types [17]. The clinical value of combined enzyme assessment has also been reported in male breast cancer, where a joint score based on pretreatment ALP and LDH separated patients into prognostic groups and remained an independent predictor of disease-free survival and overall survival in multivariable analysis [28].

From a clinical standpoint, combined preoperative ALP-LDH status offers a potentially attractive adjunct to existing risk-stratification approaches before pancreaticoduodenectomy, given that both markers are inexpensive, routinely available, and require no additional testing infrastructure even in resource-limited settings such as ours. If validated in larger cohorts, patients identified as having both markers elevated could be flagged preoperatively for closer perioperative surveillance, reinforced wound-care and infection-prevention protocols, and more detailed preoperative counselling regarding the possibility of postoperative complications. Such stratification could also inform resource allocation, for example, prioritising high-risk patients for postoperative monitoring in a higher level of care setting. However, given the exploratory nature and limitations of the present study, combined ALP-LDH status should not, at this stage, be used as a stand-alone decision-making tool for patient selection, surgical planning, or counselling; rather, it should be considered as one component within a broader preoperative risk assessment that also incorporates established clinical, radiological, and biochemical parameters (including bilirubin and CA 19-9), pending confirmation in larger, multicentre, prospective studies with adjusted analyses and survival follow-up.

This study has several limitations. The ALP and LDH cut-off values were generated from the same dataset in which their associations with postoperative outcomes were subsequently assessed. Consequently, the apparent discriminatory performance may be optimistic because no internal resampling procedure or independent validation cohort was available. The sample size was small, and the groups were markedly imbalanced, particularly Group 1 with only two patients, making one-way ANOVA assumptions difficult to verify and limiting the precision of subgroup comparisons. Moreover, ALP is closely related to biliary obstruction; the observed association may be confounded by the severity of jaundice, cholangitis, or biliary instrumentation. Total bilirubin was markedly higher in the combined-elevated group, although the between-group comparison did not reach conventional statistical significance. The inclusion of four anatomically related but biologically distinct periampullary tumour subtypes introduces clinical heterogeneity. Differences in tumour biology, biliary obstruction, nutritional status, stage distribution, and operative risk may have influenced both biomarker levels and postoperative outcomes. The sample size was insufficient for meaningful tumour subtype-stratified analyses. The small sample also did not allow reliable adjustment for these factors. The sample size calculation supported the overall cohort size but did not ensure adequate power for three unequal ALP-LDH strata. Multiple comparisons were performed without formal adjustment, so statistically significant findings should be interpreted as hypothesis-generating rather than confirmatory. The study was also single-centre, without external validation and without long-term survival follow-up.

## Conclusions

This exploratory study suggests that preoperative combined ALP-LDH status may be associated with postoperative wound infection and a non-significant trend toward higher rates of major complications. Most other postoperative outcomes were not significantly associated with ALP-LDH status. Given the small, markedly imbalanced sample and the absence of correction for multiple comparisons, these findings should be interpreted as hypothesis-generating rather than confirmatory and do not yet support use of combined ALP-LDH status as a stand-alone predictive tool. A larger, multicentre, prospective study with adjusted analyses and survival follow-up is recommended to confirm whether combined ALP-LDH status has value as a predictor of postoperative outcomes.
